# Seawater Acidification Reduced the Resistance of *Crassostrea gigas* to *Vibrio splendidus* Challenge: An Energy Metabolism Perspective

**DOI:** 10.3389/fphys.2018.00880

**Published:** 2018-07-12

**Authors:** Ruiwen Cao, Yongliang Liu, Qing Wang, Dinglong Yang, Hui Liu, Wen Ran, Yi Qu, Jianmin Zhao

**Affiliations:** ^1^Muping Coastal Environmental Research Station, Yantai Institute of Coastal Zone Research, Chinese Academy of Sciences, Yantai, China; ^2^Research and Development Center for Efficient Utilization of Coastal Bioresources, Yantai Institute of Coastal Zone Research, Chinese Academy of Sciences, Yantai, China; ^3^University of Chinese Academy of Sciences, Beijing, China

**Keywords:** ocean acidification, *Crassostrea gigas*, *Vibrio splendidus*, oxidative stress, energy metabolism, physiological response

## Abstract

Negative physiological impacts induced by exposure to acidified seawater might sensitize marine organisms to future environmental stressors, such as disease outbreak. The goal of this study was to evaluate if ocean acidification (OA) could reduce the resistance capability of the Pacific oyster (*Crassostrea gigas*) to *Vibrio splendidus* challenge from an energy metabolism perspective. In this study, the Pacific oyster was exposed to OA (pH 7.6) for 28 days and then challenged by *V. splendidus* for another 72 h. Antioxidative responses, lipid peroxidation, metabolic (energy sensors, aerobic metabolism, and anaerobic metabolism) gene expression, glycolytic enzyme activity, and the content of energy reserves (glycogen and protein) were investigated to evaluate the environmental risk of pathogen infection under the condition of OA. Our results demonstrated that following the exposure to seawater acidification, oysters exhibited an energy modulation with slight inhibition of aerobic energy metabolism, stimulation of anaerobic metabolism, and increased glycolytic enzyme activity. However, the energy modulation ability and antioxidative regulation of oysters exposed to seawater acidification may be overwhelmed by a subsequent pathogen challenge, resulting in increased oxidative damage, decreased aerobic metabolism, stimulated anaerobic metabolism, and decreased energy reserves. Overall, although anaerobic metabolism was initiated to partially compensate for inhibited aerobic energy metabolism, increased oxidative damage combined with depleted energy reserves suggested that oysters were in an unsustainable bioenergetic state and were thereby incapable of supporting long-term population viability under conditions of seawater acidification and a pathogen challenge from *V. splendidus*.

## Introduction

Increased atmospheric CO_2_ concentrations caused by anthropogenic activities have and will continue to contribute to ocean acidification (OA), as about 30% of CO_2_ is absorbed by the oceans (Sabine et al., 2004). The pH of surface seawater has already declined approximately 0.1 pH units compared with pre-industrial levels (Intergovernmental Panel on Climate Change, 2014). With further increases of atmospheric CO_2_ partial pressure (*p*CO*_2_*), surface ocean pH is predicted to decline by another 0.3–0.4 pH units by the end of this century (Caldeira and Wickett, 2005). Due to biogeochemical and biochemical processes, such as eutrophication, anthropogenic sulfur and nitrogen deposition, and the existence of a mid-salinity minimum buffer zone, pH in coastal and estuary areas is more variable than that in the open ocean (Doney et al., 2007; Cai et al., 2011; Hu and Cai, 2013).

Numerous studies have indicated that coastal ecosystems are under more serious threat from OA combined with other environmental stressors, including increased ocean temperature, pollution, and pathogenic challenge (Chen et al., 2015; Queirós et al., 2015; Gunderson et al., 2016; Moreira et al., 2016; Duckworth et al., 2017). Seawater acidification has been found to negatively affect physiological processes in mollusks such as calcification, respiration, filtration, acid–base regulation, and immune responses (Pörtner et al., 2004; Gazeau et al., 2013; Sui et al., 2015; Liu et al., 2016; Castillo et al., 2017; Zhao et al., 2017), which could lead to severe threats for their energy budget (Wang et al., 2015). As a consequence, the negative impact posed by acidified seawater might sensitize marine organisms to future environmental perturbations, such as pathogenic infection (Hooper et al., 2007; Ellis et al., 2015). However, few studies have investigated the integrated environmental impacts of OA and pathogen infection on marine organisms. *Vibrio*sis, one of the major bacterial diseases affecting fish, bivalves, and crustaceans, is caused by pathogenic *Vibrio* species and has posed a severe challenge to marine organisms inhabiting coastal and estuary areas (Beaz-Hidalgo et al., 2010). It is therefore urgently needed to investigate the environmental and ecological future of coastal and estuarine regions under acidified seawater and pathogenic *Vibrio* infection.

Marine bivalves are globally recognized as valuable species from ecological and economic perspectives (Dumbauld et al., 2009). The Pacific oyster, *Crassostrea gigas*, is an important aquaculture bivalve species inhabiting estuaries and intertidal regions, where they are constantly confronted with various environmental perturbations. As a result, oysters are believed to be highly tolerant to multiple environmental stressors (Dineshram et al., 2016). However, some strains of *Vibrio splendidus* have been associated with summer mortality of juvenile oysters *C. gigas* (Lacoste et al., 2001; Gay et al., 2004). In addition, recent studies have found that OA could lead to immune suppression of marine invertebrates, especially marine bivalves, which could sensitize these species to pathogen infection (Hernroth et al., 2011; Ivanina et al., 2014; Liu et al., 2016; Wang et al., 2016).

Previous studies have shown that both OA and pathogenic infection could induce disturbances in energy metabolism and oxidative stress in marine organisms (Canesi et al., 2010; Ellis, 2013; Liu et al., 2013; Ellis et al., 2014; Lesser, 2016). The bioenergetics sustainability under environmental perturbations is linked directly to an organism’s fitness and has thus population-level consequences (Sokolova et al., 2012). Our proteomic study has also found that energy metabolism disturbance was correlated with oyster immunosuppression in response to seawater acidification and pathogens challenge (Cao et al., 2018). As a follow-up study, antioxidant enzyme activities (CAT, SOD, and GPx) and lipid peroxidation (LPO) were investigated in *C. gigas*. Moreover, the expression responses of genes related to energy sensors (Axin1, AMPKβ, and SIRT2), aerobic metabolism [TCA cycle enzymes, electron transport chain (ETC) components, and ATP turnover], and anaerobic metabolism (D-LDH X1 and D-LDH X2) combined with energy reserve content (GLY and PROT) were also investigated to assess the energy expenditure response, and further elucidated whether the altered oyster energy metabolism strategy was long-term sustainable in response to OA and/or *V. splendidus*.

## Materials and Methods

### Ocean Acidification Perturbation Experiment

Adult Pacific oysters (8–11 cm in shell length) were collected from a local farm from Muping, Yantai, and Shandong Provinces of China (37°38′N and 121°59′E; pH 8.10 ± 0.03) in December 2016, and acclimated in the laboratory for 2 weeks before commencement of the experiment. During the acclimation period, the organisms were maintained at a temperature of 17.3 ± 0.2°C and pH 8.14 ± 0.02 in aerated seawater (salinity 31.2 ± 0.5). After the preliminary acclimation, oysters were exposed for four weeks to two levels of seawater pH (8.1 or 7.6), followed by a 72 h challenge treatments (non-injection, filter-sterilized seawater-injection, or *V. splendidus*-injection), for a total of six treatments. The selected pH levels were representative of the present-day condition (pH 8.1) and the predicted condition by the moderate scenarios of Forster et al. (2007) for the year 2250 (pH 7.6). For each treatment, 3 aquaria were used with 20 organisms per aquarium (60 individuals per treatment). The control seawater was bubbled with the atmospheric air. Seawater bubbled with air–CO_2_ mixtures was adjusted through an air and CO_2_ gas flow adjustment system and set as the elevated *p*CO_2_ treatment (pH 7.6). Oysters were fed twice daily with a commercial algal blend (containing *Chlorella vulgaris* Beij and *Phaeodactylum tricornutum*).

During the exposure period, seawater was renewed every other day using pre-equilibrated seawater. No oyster mortality was observed during OA exposure. The pH of each container was monitored daily with a pH electrode (pH meter PB-10, Sartorius Instruments, Germany) calibrated with NBS standard pH solution. Salinity, temperature, and dissolved oxygen (DO) were determined daily by using an YSI meter (YSI^®^ model 85, Yellow Springs, OH, United States). Water samples were collected from each tank every week to determine total alkalinity (TA) by potentiometric titration (Gran, 1952). Other related parameters of the carbonate chemistry were calculated according to known values of pH and TA levels using the software CO_2_SYS with the constants for seawater pH from Millero et al. (2006) and for KSO_4_^-^, from Dickson (1990). Seawater carbonate chemistry parameters of all treatments are presented in Supplementary Table S1.

### Bacterial Challenge Experiment

*Vibrio splendidus* was cultured in 2216E medium at 28°C overnight, and centrifuged at 4000 *g* for 10 min at 25°C. Then the pellet was re-suspended in filter-sterilized (0.22 μm pore size) seawater (FSSW) and adjusted to a final concentration of 5 × 10^7^CFU mL^−1^.

After 28 days of OA exposure, either 100 μL of FSSW (fssw-inj group) or 100 μL of *V. splendidus* suspension (5 × 10^6^ CFU per oyster, *Vibrio-*inj group) was injected into the adductor muscle of oysters. Oysters without injection (non-inj group) were also sampled after OA exposure. After processing, the animals from each treatment were returned to respective aquariums for another 72 h. No oyster mortality was found during the infection period.

### Sampling Procedure and Biochemical Analysis

At the end of the exposure period, six replicates of digestive glands and gills were sampled in each treatment. The tissues were immediately frozen in liquid nitrogen and stored at −80 °C until used for biochemical analyses. Meanwhile, six replicates of digestive glands were carefully excised and stored in RNAlater solution (Ambion, Austin, TX, United States) for subsequent RNA extraction.

#### Antioxidant Enzyme and Glycolytic Enzyme Activity Assay

Oyster gills were homogenized in phosphate buffer (50 mM potassium dihydrogen phosphate; 50 mM potassium phosphate dibasic; 1 mM EDTA; pH 7.0) and centrifuged at 10,000 *g* for 20 min at 4°C to obtain the supernatants. The resultant supernatants were subjected to antioxidant enzymes assays and LPO determination. Catalase (CAT) activity was determined according to the modified method of Vega-López et al. (2007), following the protocol proposed by Radi et al. (1991). This method evaluated the alteration in absorbance resulting from hydrogen peroxide (H_2_O_2_) degradation at 240 nm, with enzyme activity expressed as U mg^−1^ of PROT. Superoxide dismutase (SOD) activity was quantified using the method proposed by Beauchamp and Fridovich (1971), with some modifications adapted by Carregosa et al. (2014). One unit of SOD activity was defined as the amount of enzyme that caused 50% inhibition in the rate of nitroblue tetrazolium (NBT) chloride reduction and was expressed as U mg^−1^ of PROT. Glutathione peroxidase (GPx) activity was measured as described by Lawrence and Burk (1976). The method assessed the decrement in nicotinamide adenine dinucleotide phosphate (NADPH) concentration in an assay coupled to glutathione reductase (GR) that catalyzed NADPH oxidation at 340 nm. The results were expressed as U mg^-−^ of PROT. LPO levels were quantified by measuring the malondialdehyde (MDA) content, according to the method previously described by Ohkawa et al. (1979). MDA concentrations were measured spectrophotometrically at OD 532 nm and the results were expressed as nmol MDA mg^−1^ of PROT.

Enzymatic activities of HK and PK were determined in the digestive glands of OA and/or *V. splendidus*-exposed oysters. The activities of HK and PK were determined as described by Greenway and Storey (1999) and the results were expressed as U g^−1^ of PROT.

#### Energy Reserves

Glycogen content was measured by using the phenol-sulfuric acid method described by Yoshikawa (1959). A calibration curve was obtained by using glucose standards. PROT content was quantified following the Biuret method (Robinson and Hogden, 1940). Bovine serum albumin (BSA) was used as the standard material. The GLY and the PROT content was expressed as mg per g FW (fresh weight).

### RT-PCR Analysis

The mRNA expression levels of eight genes related to energy metabolism (ACN, IDH, SDH, SCS, COX 1, COX, AK, ATP synthase subunit alpha Axin1, AMPKβ, SIRT2, D-LDH X1, and D-LDH X2) and two immune related genes (Integrin beta-1B and TNF) were determined in OA and/or *V. splendidus* exposed treatments. Total RNA was extracted with TRIzol reagent (Invitrogen, United States) following the manufacturer’s instructions. DNase I (Promega, United States) treated RNA was used as a template for cDNA synthesis. A standard protocol was undertaken to perform quantitative real-time PCR program by using an Applied Biosystems 7500 fast Real-Time PCR System (Applied Biosystems, United States). The primers designed for quantitative RT-PCR are presented in Supplementary Table S3. A dissociation curve analysis of amplification products was performed at the end of each PCR to confirm that only one PCR product was amplified and detected. Expression of target genes was normalized with the elongation factor 1-alpha reference gene (de Lorgeril et al., 2011), which was stable in the current study (*p* > 0.05, coefficient of variation < 5%). The comparative 2^-ΔΔCT^ method was used to analyze the expression level of the selected genes (Livak and Schmittgen, 2001).

### Integrated Biomarker Response (IBR)

The “Integrated Biomarker Response version 2” (IBRv2) index was utilized to integrate multiple biochemical parameters into one general index representing the environmental stress level of different treatments (Sanchez et al., 2013). To calculate the IBRv2 values, individual biomarker data (Xi) were compared to reference data (X0) and log transformed to reduce variance: Yi = log Xi/X0. The general mean (μ) and SD of each biomarker Yi were computed for all treatments; Yi was standardized as Zi = (Yi-μ)/s. Biomarker deviation index (A) was calculated using the mean of the standardized biomarker response (Zi) and mean of reference biomarker data (Z0): Ai = Zi-Z0. For a single treatment, the biomarker deviation index (A) was presented in star plots, representing the deviation of each investigated biomarker in OA and/or *V. splendidus* exposed groups, compared to the control group. The area above 0 reflected biomarker induction, and the area below 0 indicated a biomarker inhibition. In addition, the IBRv2 was calculated for each biomarker in each treatment: IBRv2 = Σ|A|.

### Statistical Analysis

All measured biochemical parameters were presented as the mean ± SD. The raw data were assessed for normality and homogeneity of variances using the Shapiro–Wilk test and Bartlett’s test, respectively. A two-way ANOVA followed by Fisher’s least significant difference (LSD) *post hoc* analyses was performed on all data to test for significant differences (*p* < 0.05) between treatments. The analyses were carried out using SPSS version 23.0 (SPSS Inc., Chicago, IL, United States). ANOVA results for all studied parameters are displayed in Supplementary Tables S4, S5. Principal component analysis (PCA) was performed with CANOCO 5.0 software (Microcomputer Power Inc., United States) using gene expression data from OA and *V. splendidus* exposed oysters to assess the variability associated with energy metabolism and immune responses.

## Results

### Antioxidant Enzyme Responses and Oxidative Damage

**FIGURE 1:**
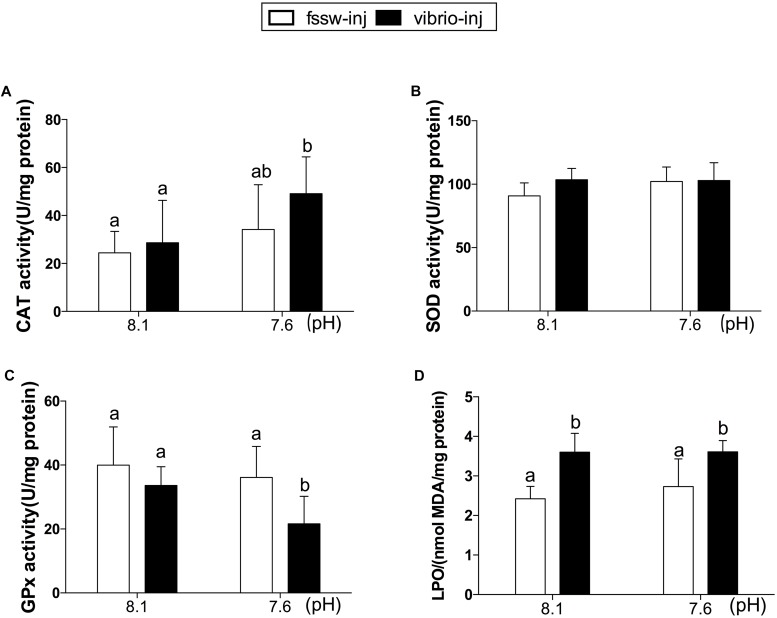
Antioxidant enzyme activities and LPO levels in the gills of *C. gigas* post elevated *p*CO_2_ and/or *V. splendidus* exposure. **(A)** CAT. **(B)** SOD. **(C)** GPx. **(D)** LPO. Each bar represents mean ± SD (*n* = 6). Different letters indicate significant differences among different treatments (*p* < 0.05).

There was no significant change in any of the physiological parameters measured in fssw-inj-treated oysters compared to non-inj-treated oysters (Supplementary Table S2). As a result, the oysters treated with fssw-injection were set as the control in the present study. The antioxidant enzyme responses and oxidative damage under OA and/or *Vibrio* exposure are shown in **Figure 1**. CAT activity increased significantly in oysters treated with *Vibrio* at pH 7.6 (*p* < 0.05, **Figure 1A**). However, no significant changes in CAT activity were observed in oysters exposed to either OA or *Vibrio* alone. Similarly, there was no significant change in SOD activity among all four treatments (Control, *Vibrio*, OA, and *Vibrio*-OA groups; **Figure 1B** and Supplementary Table S4). Regarding GPx activity, significant inhibition effect was observed in oysters under combined OA and *Vibrio* exposure (*p* < 0.05, **Figure 1C**). Oysters showed significantly higher LPO levels in response to *Vibrio* challenge, either alone or combined with OA (**Figure 1D**). However, oysters exposed only to OA showed higher but not significantly different LPO levels compared to the control individuals (**Figure 1D** and Supplementary Table S4).

### Glycolytic Enzyme Activities and Energy Reserves

**FIGURE 2:**
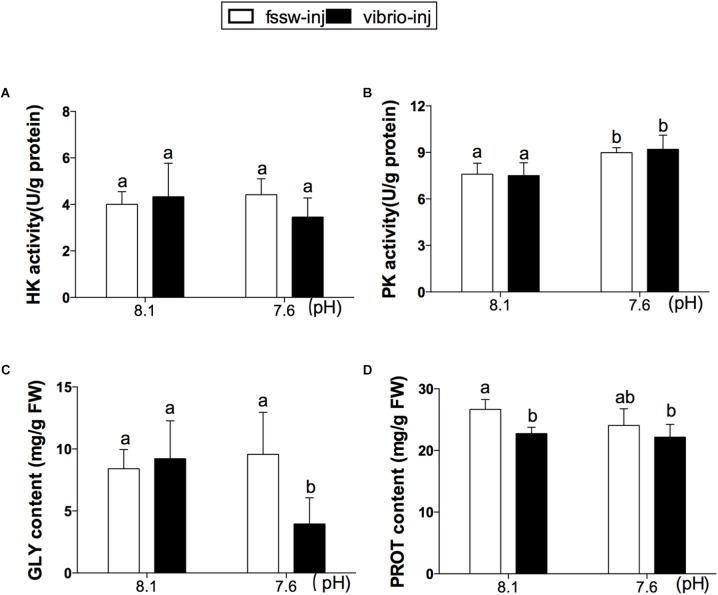
Glycolytic enzyme activities and energy reserves in the digestive glands of *C. gigas* post elevated *p*CO_2_ and/or *V. splendidus* exposure. **(A)** HK activity. **(B)** PK activity. **(C)** GLY content. **(D)** PROT content. Each bar represents mean ± SD (*n* = 6). Different letters indicate significant differences among different treatments (*p* < 0.05).

No significant difference in HK activity was found among individuals in the four treatments (**Figure 2A**). However, the enzyme activity of PK was significantly increased in OA-treated oysters, regardless of *Vibrio* challenge (*p* < 0.01; **Figure 2B** and Supplementary Table S4). Further, as a major energy reserve, GLY content was significantly decreased in response to the combined exposure of OA and *V. splendidus* compared to other treatments (*p* < 0.05; **Figure 2C** and Supplementary Table S4). No significant change in GLY content was found in the digestive glands of oysters treated with either OA or *Vibrio*. However, PROT content was significantly lower in *Vibrio* and *Vibrio*-OA treatments compared to the control group (**Figure 2D**), while no apparent change was found in OA-treated oysters compared with the other three treatments.

### Expression of Genes Related to Energy Metabolism and Immune Responses

**FIGURE 3:**
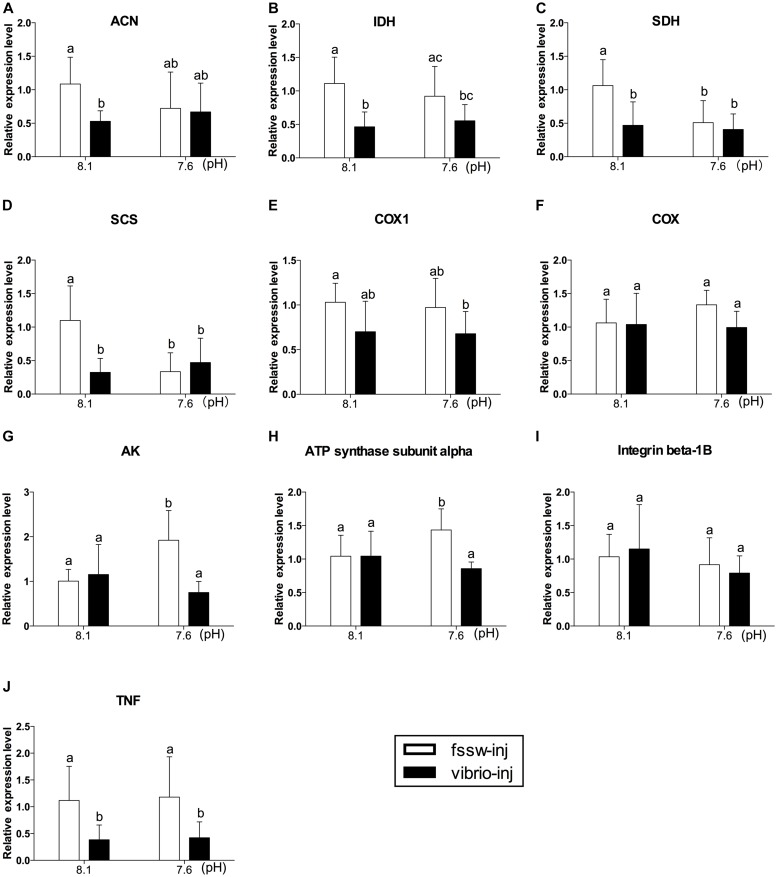
The mRNA expression profiles of aerobic energy metabolism- and immune-related genes in the digestive glands of oysters post elevated *p*CO_2_ and *V. splendidus* exposure. **(A)** ACN. **(B)** IDH. **(C)** SDH. **(D)** SCS. **(E)** COX 1. **(F)** COX. **(G)** AK. **(H)** ATP synthase subunit alpha. **(I)** Integrin beta-1B. **(J)** TNF. Each bar represents mean ± SD (*n* = 6). Different letters indicate significant differences among treatments (*p* < 0.05), and identical letters indicate no significant difference.

The expression levels of genes related to aerobic metabolism, including the TCA cycle (ACN, IDH, SDH, and SCS), mitochondria electron transfer chain components (COX1 and COX), and ATP metabolism (ATP synthase subunit alpha and AK), and two immune factors (TNF and integrin beta-1B), were investigated in oysters under exposure of OA and/or *Vibrio* (**Figure 3**). The mRNA expression of TCA cycle-related genes (ACN, IDH, SDH, and SCS) was significantly inhibited under *Vibrio* challenge (**Figures 3A–D** and Supplementary Table S5). The expression level of two TCA cycle enzyme (SDH and SCS) transcripts was significantly inhibited under OA exposure without *Vibrio* challenge compared to the control individuals (**Figures 3C,D**). To the contrary, the mRNA expression of ATP metabolism related genes (ATP synthase and AK) were significantly stimulated under OA challenge without *Vibrio* infection compared to other treatments (**Figures 3G,H**).

The expression pattern of all genes tested in *Vibrio*-OA-treated oysters was similar to that of *Vibrio*-treated individuals. The expression level of most of the aerobic metabolism-related genes (IDH, SDH, SCS, and COX1) was depressed under combined exposure to OA and *Vibrio*, but OA individually did not elevate the inhibition effect (**Figures 3A–E** and Supplementary Table S5). ANOVA results also suggest that there is generally no significant interaction between OA and *Vibrio* challenges, with the exception of SCS, ATP synthase subunit alpha, and AK (Supplementary Table S5). The mRNA expression of ATP synthase subunit alpha and AK transcripts was significantly decreased in oysters under *Vibrio*-OA exposure compared to OA treatment, suggesting an antagonistic relationship between *Vibrio* and OA on the expression of these two genes (**Figures 3G,H**). The expression level of integrin beta-1B transcripts was not altered in response to OA and *Vibrio* challenges, individually or in combination (**Figure 3I**). *Vibrio* challenge significantly inhibited the expression of TNF transcripts, regardless of OA exposure (**Figure 3J**), while no significant change was observed in oysters under OA exposure alone.

**FIGURE 4:**
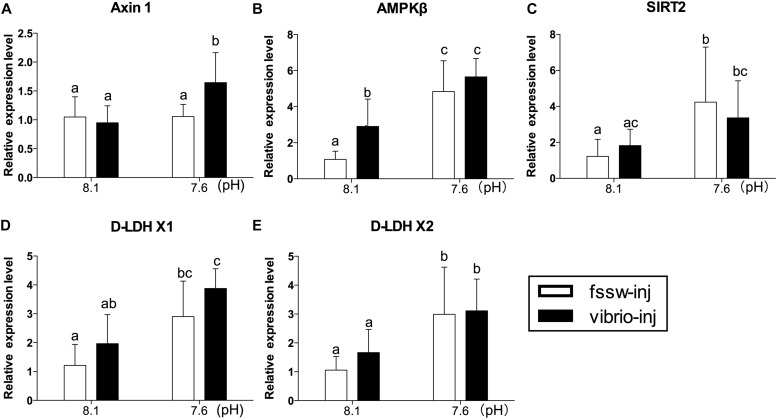
The mRNA expression profiles of anaerobic energy metabolism- and energy sensing-related genes in the digestive glands of oysters post elevated *p*CO_2_ and *V. splendidus* exposure. **(A)** Axin1. **(B)** AMPKβ. **(C)** SIRT2. **(D)** D-LDH X1. **(E)** D-LDH X2. Each bar represents mean ± SD (*n* = 6). Different letters indicate significant differences among treatments (*p* < 0.05), and identical letters indicate no significant difference.

The expression responses of genes related to metabolic sensors (Axin1, AMPKβ, and SIRT2) and anaerobic glycolysis (D-LDH X1 and D-LDH X2) were also investigated. Our results showed significantly elevated expression of all genes (Axin1, AMPKβ, SIRT2, D-LDH X1, and D-LDH X2) investigated under exposure to *Vibrio* and OA (**Figures 4A–E** and Supplementary Table S5). A significant stimulation effect was observed in the expression of AMPKβ transcripts of *Vibrio*-treated oysters (**Figure 4B**). In oysters treated with OA alone, the expression of all genes (AMPKβ, SIRT2, D-LDH X1, and D-LDH X2), with the exception of Axin1, was significantly elevated compared to the control group (**Figures 4B–E**). However, a significant interaction between OA and *Vibrio* was observed only in the expression of Axin1 transcripts (Supplementary Table S5).

### Principal Component Analysis (PCA)

**FIGURE 5:**
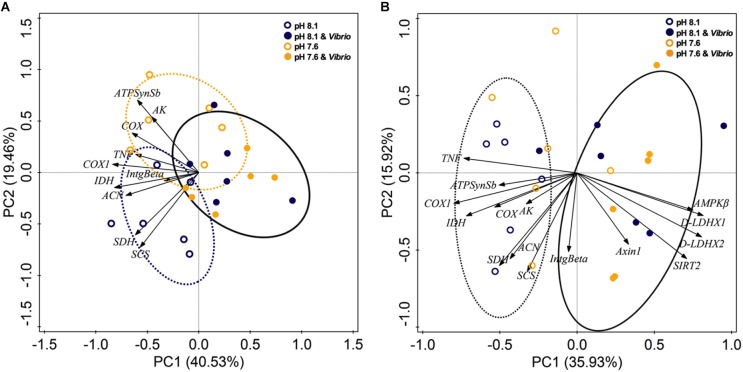
**(A)** Principle component analysis (PCA) ordination biplot of ocean acidification and/or *V. splendidus* treatments for *C. gigas*, using expression data of genes related to aerobic metabolism and immune responses. **(B)** PCA ordination biplot of ocean acidification and/or *V. splendidus* treatments for *C. gigas*, using expression data of genes related to energy metabolism and immune responses.

Principal component analysis was applied to all expression abundance data of aerobic energy metabolism-related genes and two immune genes from digestive glands (**Figure 5A**) and revealed 59.99% of the total variance. PC1 represented 40.53% of variance, highlighting the separation between non- and *Vibrio* exposed oysters. The second component (explaining only 19.46% of the variance) identified the separation of non-OA and OA exposure treatments without *Vibrio* challenge. Overall, PCA analysis provided further validation for the clustering of aerobic energy metabolism-related genes on the negative side of PC 1. Furthermore, PC 1 showed the clustering of *Vibrio* treatments in opposition to the expression elevation of all measured aerobic metabolism-related genes.

Additionally, PCA clustering of expression abundance data of energy metabolism-related (metabolic sensors, aerobic metabolism, and anaerobic metabolism) genes and two immune genes showed a total 51.85% variance (**Figure 5B**). PC1 expressed 35.93% variance, revealing separation between non- and *Vibrio-* exposed treatments. *Vibrio* and *Vibrio*-OA treatments correlated positively with metabolic sensor- and anaerobic metabolism-related genes and correlated negatively with aerobic metabolism-related genes. These results clearly demonstrated that in response to *Vibrio* challenge, either alone or combined with OA, oysters exhibited suppressed aerobic metabolism and stimulated aerobic metabolism.

### Integrated Biomarker Response (IBR)

**FIGURE 6:**
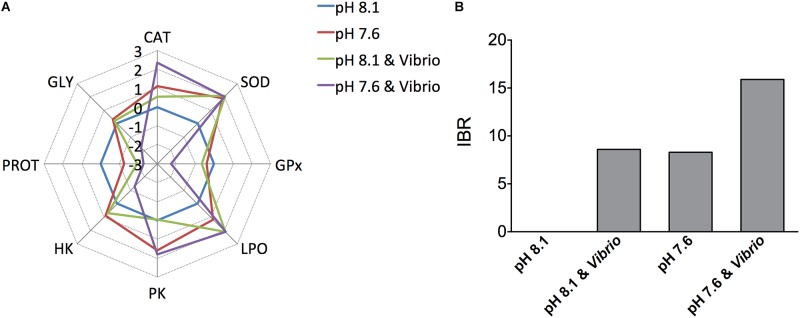
Biomarker star plots and integrated biomarker response (IBR). **(A)** Biomarker star plots for each treatment displaying the general patterns of data variability. SOD, superoxide dismutase; CAT, catalase; GPx, glutathione peroxidase; LPO, lipid peroxidation; PK, pyruvate kinase; HK, hexokinase; PROT, protein; GLY, glycogen. **(B)** Calculated IBR index using the biochemical parameters measured in *C. gigas* after exposure to ocean acidification and/or *V. splendidus*.

The IBR star plots present transformed data for each measured biochemical parameter (antioxidant enzymes and oxidative damage, glycolytic enzymes, and energy reserves) in oysters exposed to an OA and *Vibrio* challenge (**Figure 6A**). Star plots revealed that most of the parameters (CAT, GPx, HK, PROT, and GLY) showed higher response in oysters exposed to both *Vibrio* and OA than single stressor-treated individuals. The higher IBR index in oysters treated with *Vibrio*-OA, compared to single stressor-treated individuals, suggested that oysters were under severe stress in response to the combined exposure of *Vibrio* and OA. The integrated biomarker response (IBR) values in each treatment are shown in **Figure 6B**. The IBR index was similar between the OA and *Vibrio* treatments, while the IBR index was exceedingly high in *Vibrio*-OA treatment compared to either the OA or *Vibrio* treatments alone. According to the IBR index, the rank order of treatments was: *Vibrio*-OA > *Vibrio* = OA.

## Discussion

### Antioxidant Responses and Oxidative Damage

ROS are naturally produced and maintained at basal levels during cellular aerobic metabolism (Halliwell and Gutteridge, 1986). Numerous environmental stressors have been found to induce ROS production in marine invertebrates (Canesi et al., 2010; Tomanek et al., 2011; Zhu et al., 2011; Matoo et al., 2013; Ferreira et al., 2015). LPO, caused by excessive ROS production, is a major indicator of oxidative damage in organisms and a major contributor to the loss of cell function under environmental stress, including OA and pollutants (Moreira et al., 2016; Ricevuto et al., 2016; Valerio-García et al., 2017). In the present study, the LPO level increased significantly in *Vibrio*-treated oysters. Similarly, increased oxidative stress and altered antioxidant enzyme responses were observed in *Mytilus galloprovincialis* challenged with *V. splendidus* (Canesi et al., 2010). It has been shown that the adrenergic receptors and PKC-mediated pathways’ activation induced by bacterial components were associated with ROS production (Dailianis et al., 2005; Ciacci et al., 2010). Meanwhile, antioxidant enzymes such as CAT and SOD were shown to remove excessive ROS in organisms under severe stress caused by environmental perturbations (Giarratano et al., 2014). Although the LPO level increased significantly in *V. splendidus* challenged oysters, no significant change was observed in the antioxidant enzyme activities (CAT, SOD, and GPx) in this study. These results suggest that inefficient activation of antioxidant defenses in *Vibrio*-treated oysters resulted in higher oxidative damage in these individuals compared to those in the control group.

However, the present findings demonstrated that oysters were able to increase the activities of antioxidant enzymes, such as CAT, to counteract the oxidative stress caused by combined exposure to OA and *Vibrio*. In contrast, the activity of GPx decreased significantly in the *Vibrio*-OA treatment. The inhibited responses of the antioxidant enzymes possibly suggested a failure of the antioxidant system in scavenging ROS, and thus resulted in the accumulation of the oxidative substance in the cells (Li et al., 2012). Accordingly, LPO level was increased significantly in response to the combined exposure to OA and *V. splendidus*.

Oxidative stress has been suggested to be involved in the toxic mechanism of seawater acidification (Benedetti et al., 2016; Moreira et al., 2016; Velez et al., 2016a). For example, a proteomic study on eastern oysters, *Crassostrea virginica*, suggested an induced oxidative stress after a 2-week exposure to elevated CO_2_ concentration (∼357 Pa *p*CO_2_; Tomanek et al., 2011). However, our results revealed that OA did not stimulate antioxidant responses and oxidative damage in oysters, indicating a low oxidative stress level in OA-exposed oysters. Similar results have also been found in two bivalve species, *C. virginica* and *Mercenaria mercenaria*, in which no persistent oxidative stress signal was observed during long-term exposure to OA (∼800 ppm CO_2_; Matoo et al., 2013).

In general, although OA alone did not induce significant changes in antioxidant enzyme activities, the combined exposure to OA and *Vibrio* led to higher oxidative stress compared to that of each stressor alone. This outcome was demonstrated by significantly altered antioxidant enzyme activities (CAT and GPx) and was expected as both OA exposure and *V. splendidus* challenge could lead to increased ROS production in oysters (Canesi et al., 2010; Tomanek et al., 2011). Above all, the results from this study demonstrated that excessive ROS production in oysters exposed to *Vibrio* alone or combined with an OA challenge induced cellular stress and oxidative damage and contributed therefore to the pathogenesis of *Vibriosis*.

### Aerobic Metabolism and Energy Reserves

Energy metabolism biomarkers were vital for evaluating cellular energy status under environmental perturbations, and could be used to predict ecological consequences of stress exposure (Dong and Zhang, 2015). Under normal conditions, ATP supply via aerobic metabolism was sufficiently high to cover the maintenance costs, as well as energy costs of physiological activity, growth, and reproduction. However, severe environmental stress could lead to depressed aerobic metabolism in invertebrates (Giacomin et al., 2014; Dahlke et al., 2016; Zhao et al., 2017). Hence, the expression levels of aerobic metabolism-related genes were investigated in this study. Specifically, the TCA cycle served as the most important phase in respiratory process, oxidizing acetate into carbon dioxide (Fernie et al., 2004). ACN, IDH, SDH, and SCS are essential enzymes that participate in the mitochondrial TCA cycle. COX1 and COX are components of the mitochondria ETC (Wikstrom, 1977). Two ATP metabolic genes, including ATP synthase subunit alpha and AK, were also investigated in the oyster *C. gigas*.

A growing body of evidence has suggested that OA could disrupt the energy balance of mollusks by impairing key processes associated with high energy expenditure, such as immunity, development, acid–base homeostasis maintenance, and antioxidant processes (Lannig et al., 2010). A disturbance in energy metabolism by OA could sensitize bivalves to other environmental perturbations such as pathogen infection (Ivanina and Sokolova, 2015; Hernroth et al., 2016). In this study, the suppressed expression of two genes (SDH and SCS) in OA-treated oysters suggested only a mild inhibition effect of OA on aerobic energy metabolism. The increased mRNA expression of ATP synthase subunit alpha and AK might be associated with increased ATP turnover rates in OA treatment, which suggested a partial energy compensation for stress response in OA-treated oysters. In addition, enhanced glycolysis, indicated by increased PK activity, was observed in OA-treated oysters, which may supply the energy needed for acid–base homeostasis maintenance and other stress responses in oysters under OA exposure. The two energy reserves (GLY and PROT) were not altered in oysters under OA exposure, suggesting the maintenance of energy homeostasis in this treatment. Above all, our results indicated that OA-exposed oysters might exploit increased glycolysis and ATP turnover to provide the energy needed for cellular homeostasis, thereby explaining the high resistance of this species to OA.

From our findings, *V. splendidus* challenge was the major factor affecting oyster energy metabolism. Aerobic energy metabolism was decreased in *Vibrio* and *Vibrio*-OA treatments, which was demonstrated by the significantly inhibited expression of most aerobic energy metabolic genes. PCA analysis further confirmed the inhibition effect on the expression of aerobic metabolism-related genes caused by *Vibrio* exposure, either alone or combined with OA (**Figure 5A**). Although aerobic metabolism was inhibited, decreased PROT content in oysters treated by *Vibrio* alone or combined with OA and decreased GLY levels in the *Vibrio*-OA treatment suggested a disturbed energy balance. In addition, PK catalyzes the final step of glycolysis, which plays an important role in GLY metabolism (Hemre et al., 2002). Increased PK activity found in the present study indicated enhanced levels of glycolysis in *Vibrio*-OA treatment. Similarly, GLY has been mobilized as a source of energy for cellular protection mechanisms under extreme salinities (Velez et al., 2016b).

### Energy Sensors and Anaerobic Metabolism

Energy homeostasis in invertebrates could be maintained through cellular pathways involved in cellular energy status sensing and energy expenditure modulation (Han et al., 2013; Dong et al., 2014; Dong and Zhang, 2015; Jost et al., 2015). When cellular energy status was compromised, activation of AMPK was initiated by assembling the Axin-AMPK-serine-threonine liver kinase B1 (LKB1) complex (Zhang et al., 2013). Histone/PROT deacetylase (SIRT) served as another metabolic sensor in cells under low energy status. The activation of energy metabolism sensors including Axin, AMPK, and SIRT could switch on the catabolic pathways (primarily lipid and glucose catabolism), resulting in ATP generation while simultaneously shutting down ATP consuming anabolic pathways (Cantó et al., 2009; Houtkooper et al., 2012; Hardie et al., 2016). In this study, the observed significantly elevated expression levels of metabolic sensors (Axin1, AMPKβ, and SIRT2) in oysters exposed to OA and/or *Vibrio* indicated activated catabolism and inhibited anabolic metabolism. This result could further explain the increased glycolytic enzyme activities found in *Vibrio*- and/or OA-treated oysters, and depleted GLY level in oysters treated by *Vibrio* alone or combined with OA. Positive correlation between the expression of metabolic sensing genes (Axin, AMPK, and SIRT) and glycolysis-related genes has also been observed in limpets, *Cellana toreuma* (Han et al., 2013; Dong et al., 2014).

Furthermore, D-LDH has the ability to catalyze the NADH-dependent interconversion of pyruvate and D-lactate in anabolic and catabolic pathways (Simon et al., 1989). In the present study, increased expression of D-LDH X1 and D-LDH X2 observed in OA and/or *Vibrio* exposed oysters verified our hypothesis that enhanced anaerobic glycolysis was initiated to compensate for depressed energy supplies caused by inhibition of aerobic metabolism. PCA results also indicated that metabolic sensors (Axin1, AMPKβ, and SIRT2) and anaerobic metabolism (D-LDH X1 and D-LDH X2) were negatively correlated with aerobic metabolic genes in *Vibrio*-treated oysters, regardless of OA.

While the metabolic rate and oxygen consumption were not measured in the present study, previous studies showed that OA could lead to succinate accumulation in both oyster gills and hepatopancreas, as measured by NMR-base metabolomics (Lannig et al., 2010), suggesting partial anaerobiosis of oysters under OA exposure. Furthermore, decreased oxygen consumption and metabolic rate were found in OA-exposed mussel *M. galloprovincialis* (Michaelidis et al., 2005). Michaelidis et al. (2005) also found that respiratory acidosis in the extracellular fluids of mussels might be associated with the reduction in aerobic scope and aerobic metabolism. In the meantime, reduction in energy reserves within bacterial challenged bivalve species has been observed in numerous studies (Dittman et al., 2001; Paillard et al., 2004; Flye-Sainte-Marie et al., 2007). Correspondingly, OA posed milder suppression on the aerobic metabolism and led to partial anaerobiosis in oysters, while bacterial challenge alone or combined with OA could lead to suppressed aerobic metabolism, partial anaerobiosis, and decreased energy reserves in oyster *C. gigas.* In addition, severely depleted energy reserves of *V. splendidus* challenged oysters under decreased pH might be associated with higher energy cost for the acid–base hemostasis maintenance and antioxidant responses.

### Correlation of Energy Metabolism With Oxidative and Immune Response

Generally, oysters could suppress aerobic energy metabolism to decrease ROS production. Additionally, the suppressed energy metabolism and milder depleted energy reserves in *Vibrio*- or OA-treated oysters might be associated with the unaltered antioxidant enzyme response in these two treatments, as energy was mobilized to more urgent cellular stress responses, such as acid–base homeostasis and immune responses. Similarly, previous research has identified increased acid–base homeostasis maintenance under OA (Wang et al., 2017) and stimulated oxidative stress and immune responses under *V. splendidus* challenge in invertebrates (Canesi et al., 2010; Liu et al., 2013; Li et al., 2017). According to our previous proteomic results, a tradeoff between oxidative stress and energy metabolism was adopted by oysters in *Vibrio*-OA treatment (Cao et al., 2018). The ETC in mitochondria is a major site of ROS production (Chandel and Schumacker, 2000). In the *Vibrio*-OA group, both decreased abundance of ETC-related PROT expression found in our previous research and decreased mRNA expression of ETC-related gene COX1 in this study might suggest that a tradeoff exists between oxidative stress and energy metabolism.

In addition, as an immune factor, TNF played important roles in the immune functions of oysters. In this study, immune responses in oysters were perhaps suppressed as indicated by the significantly decreased expression of TNF transcripts. The depressed immune function in *Vibrio*-treated oysters could be associated with the disturbed energy metabolism caused by this treatment. Previous finding also suggested that *V. splendidus* challenge could lead to immunosuppression of marine invertebrates (Lacoste et al., 2001; Duperthuy et al., 2010).

### Integrated Biomarker Response

The IBR index provides a global view of environmental stress by combining various biomarker signals (Beliaeff and Burgeot, 2002). Numerous laboratory experiments have adopted IBR index to better review and compare results between treatments (Qu et al., 2014; Xu et al., 2016; Valerio-García et al., 2017; Wang et al., 2018). Higher IBR values indicated more stressful conditions as the IBR index was considered to be a general description of “health status” (Leiniö and Lehtonen, 2005). In the present study, oysters exposed to the combination of both stressors displayed a greatly increased IBR value compared to each stressor alone. These results demonstrated that co-exposure is the most stressful condition, which was in coordination with the results that co-exposure induced more severe responses of oxidative stress and depleted energy reserves.

Recently, an energy metabolism model has been proposed by Sokolova et al. (2012), in which transition to partial anaerobiosis and metabolic rate depression are characteristics of organisms on the pessimum range of energy metabolism. As the balance of ATP supply and demand is disrupted in the pessimum range of energy metabolism, populations are unable to survive. Although OA-exposed oysters could maintain energy metabolism homeostasis in this study, the disturbed energy metabolism (decreased aerobic metabolism and increased anaerobic metabolism) led to decreased energy reserves under pathogen infection alone or combined with OA exposure, which was energetically unsustainable in the long run. Furthermore, OA might reduce the ability of oysters to resist infection by the pathogen *V. splendidus*, as indicated by the higher IBR value of combined exposure compared to a single stressor. As a consequence, marine bivalves are under severe threat from OA caused by global climate change and pathogen infection.

## Conclusion

**FIGURE 7:**
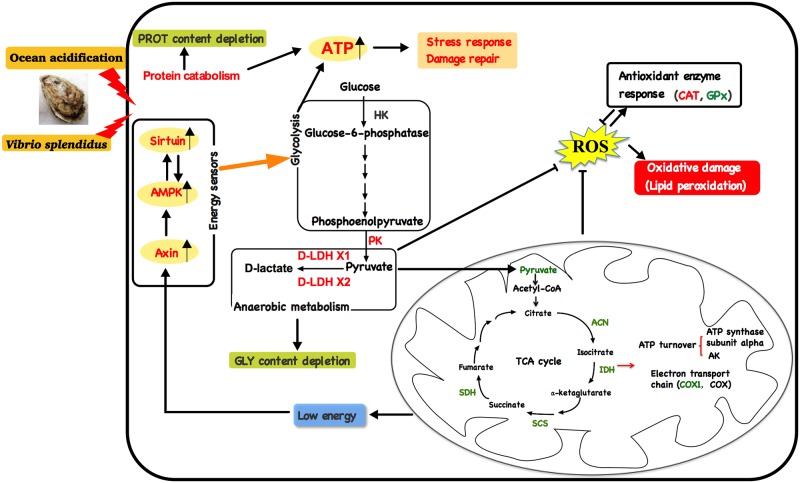
A schematic illustration showing the action of energy metabolism and oxidative stress in oysters exposed to *Vibrio* and ocean acidification.

A schematic representation was developed to conclude the oxidative and energy metabolism responses of oysters exposed to OA and *Vibrio* (**Figure 7**). In brief, OA and *Vibrio* challenges could lead to suppressed aerobic metabolism, increased anaerobic metabolism, and oxidative stress. Compromised aerobic metabolism would lead to a low cellular energy condition, which could induce increased expression of metabolic sensors. In turn, anaerobic glycolysis and other catabolic metabolisms were enhanced to provide energy for cellular stress responses and damage repair, resulting in depleted energy reserves (GLY). PROT catabolism also increased to provide additional energy under stressful conditions, thereby causing depleted PROT levels. The increased oyster energy expenditure, in response to pathogen challenge along with exposure to acidified seawater, was expected, as important physiological processes such as immune responses, antioxidant responses, and acid–base homeostasis maintenance were stimulated. Above all, our results emphasize the deleterious effects of OA and pathogen infection on cellular stress and maintenance of metabolic homeostasis in marine invertebrates.

## Author Contributions

RC and JZ conceived and designed the experimental plan, analyzed the data, and drafted the manuscript. RC, YL, QW, DY, HL, WR, and YQ performed the experiments.

## Conflict of Interest Statement

The authors declare that the research was conducted in the absence of any commercial or financial relationships that could be construed as a potential conflict of interest.

## Abbreviations

ACN: aconitate hydratase
AK: adenylate kinase
AMPK: AMP-activated protein kinase
Axin: Fu gene inhibition axis formation
COX: cytochrome c oxidase
COX 1: subunit 1 of cytochrome c oxidase
D-LDH X1: D-lactate dehydrogenase transcript variant X1 (mitochondrial)
D-LDH X2: D-lactate dehydrogenase transcript variant X2 (mitochondrial)
GLY: glycogen
HK: hexokinase
IDH: isocitrate dehydrogenase
PK: pyruvate kinase
PROT: protein
SCS: succinyl-CoA synthetase alpha subunit
SDH: succinate dehydrogenase
SIRT2: NAD-dependent deacetylase sirtuin-2
