# Supplementary material for: Seawater Acidification Reduced the Resistance of Crassostrea gigas to Vibrio splendidus Challenge: An Energy Metabolism Perspective
Source: Front Physiol. 2018 Jul 12;9:880. doi: 10.3389/fphys.2018.00880 (PMC6052255; doi:10.3389/fphys.2018.00880)
Supplement: Supplementary file 1 [file Table_1.docx]

Supplementary materials

**Seawater acidification reduced the resistance of *Crassostrea gigas* to *Vibrio splendidus* challenge: an energy metabolism perspective**

Ruiwen Cao^1,2,3^, Yongliang Liu ^1^, Qing Wang^1,2^, Dinglong Yang^1,2^, Hui Liu^1,2^, Wen Ran^1,2,3^, Yi Qu^1,2,3^, Jianmin Zhao^1,2^ ^[[1]](#footnote-1)^

^1^ Muping Coastal Environmental Research Station, Yantai Institute of Coastal Zone Research, Yantai Shandong 264117, P. R. China

^2^ Research and Development Center for efficient Utilization of Coastal Bioresources, Yantai Institute of Coastal Zone Research, Chinese Academy of Sciences, Yantai Shandong 264003, P. R. China

^3^ University of Chinese Academy of Sciences, Beijing 100049, P.R. China

**Table S1.** Measured and calculated seawater chemistry parameters during the experimental exposures. The pH was monitored daily with a pH electrode calibrated with NBS Standard pH solutions, and total alkalinity (TA) was determined weekly. *p*CO_2_, total dissolved inorganic carbon (DIC) were calculated using CO_2_SYS software.

| Parameter | Temperature  (°C) | Salinity  (‰) | pH  (NBS scale) | TA  (µmol/kg) | *p*CO_2_  (ppm) | DIC  (µmol/kg) |
| --- | --- | --- | --- | --- | --- | --- |
| pH 8.1 | 13.2 ± 0.5 | 31.2 ± 0.5 | 8.11 ± 0.02 | 2317.13±45.18 | 482.39±9.65 | 2155.70±43.07 |
| pH 7.6 | 12.7 ± 0.7 | 31.2 ± 0.5 | 7.57 ± 0.05 | 2349.39±55.19 | 1862.41±44.08 | 2368.28±56.06 |

**Table S2.** Detailed data and statistical analysis of measured physiological parameters in oysters injected with seawater, *Vibrio splendidus* and oysters that were not injected. Data of the physiological parameters were presented as mean ± SD, different letters indicated significant difference among treatments (*p* < 0.05).

|  |  | **non-inj** | **fssw-inj** | **vibrio-inj** |
| --- | --- | --- | --- | --- |
| **SOD** | pH 8.1 | 102.34 ± 9.74^a^ | 90.86 ± 10.25^a^ | 103.61 ± 8.84^a^ |
|  | pH 7.6 | 94.09 ± 8.52^a^ | 102.25 ± 11.31^a^ | 102.99 ± 13.92^a^ |
| **CAT** | pH 8.1 | 24.14 ± 6.76^a^ | 24.43 ± 8.93^a^ | 28.65 ± 17.65^a^ |
|  | pH 7.6 | 27.07 ± 7.62^a^ | 34.16 ± 18.66^ab^ | 49.12 ± 15.34^b^ |
| **GPx** | pH 8.1 | 39.84 ± 8.51^a^ | 39.99 ± 11.94^a^ | 33.64 ± 5.81^a^ |
|  | pH 7.6 | 36.59 ± 9.77^a^ | 36.11 ± 9.73^a^ | 21.62 ± 8.58^b^ |
| **MDA** | pH 8.1 | 2.53 ± 0.35^a^ | 2.42 ± 0.31^a^ | 3.6 ± 0.47^b^ |
|  | pH 7.6 | 2.45 ± 0.24^a^ | 2.73 ± 0.7^a^ | 3.61 ± 0.28^b^ |
| **PK** | pH 8.1 | 7.55 ± 0.76^a^ | 7.6 ± 0.7^a^ | 7.51 ± 0.82^a^ |
|  | pH 7.6 | 8.39 ± 0.37^b^ | 8.99 ± 0.32^b^ | 9.2 ± 0.91^b^ |
| **HK** | pH 8.1 | 4.08 ± 0.64^a^ | 4.01 ± 0.54^a^ | 4.33 ± 1.44^a^ |
|  | pH 7.6 | 4.19 ± 1.13^a^ | 4.42 ± 0.68^a^ | 3.46 ± 0.82^a^ |
| **GLY** | pH 8.1 | 8.52 ± 2.04^a^ | 8.4 ± 1.55^a^ | 9.22 ± 3.04^a^ |
|  | pH 7.6 | 9.25 ± 3.41^a^ | 9.57 ± 3.39^a^ | 3.96 ± 2.10^b^ |
| **PROT** | pH 8.1 | 27.31 ± 2.17^a^ | 26.67 ± 1.61^a^ | 22.73 ± 1.03^b^ |
|  | pH 7.6 | 24.14 ± 6.64^ab^ | 24.06 ± 2.69^ab^ | 22.16 ± 2.07^b^ |

ypertrophydg tubules.ugh mantle**Table S3.** Primers used in this study.

|  | **Gene name** | **Forward primer (5'-3')** | **Reverse primer (5’-3’)** | **Genbank accession number** |
| --- | --- | --- | --- | --- |
| Reference gene | Elongation factor 1 alpha (EF1α) | CCTTTCGCAAAATGGGACG | CGGGCAACTTCTGGGACTG | AB122066.1 |
| Key genes involved in TCA cycle process | Aconitate hydratase (ACN) | CGGAAATCTCCCAGTCTGTGAA | ATCAGGTCTGCTCCCAAACAC | [XM_011444074.2](https://www.ncbi.nlm.nih.gov/nuccore/XM_011444074.2) |
|  | Isocitrate dehydrogenase (NAD+) (IDH) | CGGGAAATTACGGGGAGTTG | GACCCTTTCCTATTGGTGTTGC | [XM_011458898.2](https://www.ncbi.nlm.nih.gov/nuccore/XM_011458898.2) |
|  | Succinate dehydrogenase (SDH) | GATAAGAGCAAGTTTTGGGAAGG | GGTGAGGTGAAAGAGGTCGGT | [XM_011434595.1](https://www.ncbi.nlm.nih.gov/nuccore/XM_011434595.1) |
|  | Succinyl-CoA synthetase alpha subunit (SCS) | GCTTCCGTCATCTATGTGCCA | GGACAGCTTCATAGGTCAGGGTT | [XM_020063945.1](https://www.ncbi.nlm.nih.gov/nuccore/XM_020063945.1) |
| Component of the respiration chain in mitochondrial | Cytochrome c oxidase subunit 1 (COX1) | GTGGCTGGAATGGATATTGATACG | CTCTTGATAGAATAAGTCCTGTAAGACCC | [XM_020066397.1](https://www.ncbi.nlm.nih.gov/entrez/viewer.fcgi?db=nucleotide&id=1139750554) |
|  | Cytochrome c oxidase (COX) | TCTTGATGGCATTGATGTTCC | ATGGACCAGGTGGGCTTGA | [XM_011456187.2](https://www.ncbi.nlm.nih.gov/nuccore/1139837102) |
| ATP synthase | ATP synthase subunit alpha | AGAGAAGTGGCAGCTTTCGCTCAGTTTGG | TTAGCATCTGTGGCCTCTGTGATTTGTCC | XM_011447858.2 |
| ATP metabolic process | Adenylate kinase 2 (AK2) | GCCACCGAAAGTAAACGCTG | AGTTCTGACCCCGAGGCTAT | XM_011417129.2 |
| Energy sensing | Axin1 | CGCCGCATGTGTCTTTGATG | CAGAACACTGTGGTGATGCAG | XM_011437299.2 |
|  | AMPKβ | TTAGTTGACGGGCAGTGGGTG | TGCCAGTGCCTCAAACACCT | XM_011452145.2 |
|  | SIRT2 | AGAGATGGTTGTGAGGGGACT | TTTGACGAAGCTCATCTCCCC | XM_011449843.2 |
| Anaerobic metabolism | D-LDH X1 | AACCGGGTGGCTTTACTTGCT | GGGTCCATGGTCTGCTTGAGT | XM_020069100.1 |
|  | D-LDH X2 | AGGTCCAATGGTGGGACATGTTG | GAAGCAGCTCTCGCTTTCCG | XM_011435581.2 |
| Immune factors | Integrin beta-1B | CCTCGTAAAGAGCAGGGATG | CCATTGAGTTTGAGAGGTCCAT | AB066348.1 |
|  | Tumor necrosis factor (TNF) | AGACTGGGAGGATGTCCTGGAG | GTCAAAAACTTCTGGCTGTCAC | JH816585.1 |

**Table S4.** Two-way ANOVA: Effects of elevated *p*CO_2_ and *V. splendidus* exposure on the antioxidant enzyme activities, lipid peroxidation, glycolytic enzyme activities and energy reserves in *C. gigas*. Significant effects were highlighted in bold.

|  | | Factors/interaction | | |
| --- | --- | --- | --- | --- |
|  |  | *V. splendidus* | CO_2_ | CO_2_×*V. splendidus* |
| Enzyme activities and glycogen content | CAT | F (1, 20) =2.264  P = 0.1480 | **F (1, 20) = 5.612**  **P = 0.0280** | F (1, 20) = 0.7085  P = 0.4099 |
|  | SOD | F (1, 20) = 2.161  P = 0.1571 | F (1, 20) = 1.376  P = 0.2545 | F (1, 20) = 1.714  P = 0.2053 |
|  | GP_X_ | **F (1, 20) = 6.682**  **P = 0.0187** | F (1, 20) = 3.895  P = 0.0640 | F (1, 20) = 1.018  P = 0.3263 |
|  | MDA | **F (1, 20) = 28.45**  **P < 0.0001** | F (1, 20) = 0.6672  P =0.4236 | F (1, 20) =0.6047  P = 0.4459 |
|  | PK | F (1, 20) = 0.08316  P = 0.7760 | **F** **(1, 20) = 29.01**  **P** **< 0.0001** | F (1, 20) = 0.2087  P = 0.6527 |
|  | HK | F (1, 20) = 0.6355  P = 0.4352 | F (1, 20) = 0.3380  P = 0.5678 | F (1, 20) = 2.602  P = 0.1232 |
|  | Glycogen content | F (1, 20) = 4.984  P =0.0372 | F (1, 20) = 3.642  P = 0.0708 | **F (1, 20) = 8.964**  **P = 0.0072** |
|  | Protein content | **F (1, 20) =13.43**  **P = 0.0015** | F (1, 20) =3.976  P = 0.0600 | F (1, 20) =1.655  P = 0.2130 |

**Table S5.** Two-way ANOVA: Effects of elevated *p*CO_2_ and *V. splendidus* exposure on the expression of genes related to aerobic energy metabolism and immune response-related genes in *C. gigas*. Significant effects were highlighted in bold.

|  | | Factors/interaction | | |
| --- | --- | --- | --- | --- |
|  |  | *V. splendidus* | CO_2_ | CO_2_×*V. splendidus* |
| Energy metabolism | ACN | F (1, 20) =3.347  P = 0.0823 | F (1, 20) =0.4557  P = 0.5074 | F (1, 20) =2.290  P = 0.1459 |
|  | IDH | **F (1, 20) =13.36**  **P = 0.0016** | F (1, 20) =0.1364  P = 0.7158 | F (1, 20) =1.021  P = 0.3243 |
|  | SDH | **F (1, 20) =6.708**  **P = 0.0175** | **F (1, 20) =5.230**  **P = 0.0332** | F (1, 20) =3.350  P = 0.0821 |
|  | SCS | **F (1, 20) =4.655**  **P = 0.0433** | **F (1, 20) =4.423**  **P = 0.0483** | **F (1, 20) =9.583**  **P = 0.0057** |
|  | COX1 | **F (1, 20) =7.060**  **P = 0.0151** | F (1, 20) =0.1155  P = 0.7375 | F (1, 20) =0.025  P = 0.8770 |
|  | COX | F (1, 20) =3.115  P = 0.0928 | F (1, 20) =1.722  P = 0.2043 | F (1, 20) =0.241  P = 0.6287 |
|  | ATP synthase subunit alpha | **F (1, 20) =5.729**  **P = 0.0266** | F (1, 20) =0.7331  P = 0.4020 | **F (1, 20) =5.781**  **P = 0.0260** |
|  | AK | **F (1, 20) =6.150**  **P = 0.0222** | F (1, 20) =1.541  P = 0.2289 | **F (1, 20) =10.16**  **P = 0.0046** |
|  | Axin1 | F (1, 20) = 2.720  P = 0.1147 | **F (1, 20) = 5.718**  **P = 0.0267** | **F (1, 20) = 5.467**  **P = 0.0299** |
|  | AMPKβ | **F (1, 20) = 6.502**  **P = 0.0191** | **F (1, 20) = 39.34**  **P < 0.0001** | F (1, 20) = 0.9564  P = 0.3398 |
|  | SIRT2 | F (1, 20) = 0.03044  P = 0.8633 | **F (1, 20) = 8.124**  **P = 0.0099** | F (1, 20) = 0.8534  P = 0.3666 |
|  | D-LDH X1 | **F (1, 20) = 5.109**  **P = 0.0351** | **F (1, 20) = 22.11**  **P = 0.0001** | F (1, 20) = 0.08371  P = 0.7753 |
|  | D-LDH X2 | F (1, 20) = 0.6702  P = 0.4226 | **F (1, 20) = 14.38**  **P = 0.0011** | F (1, 20) = 0.2992  P = 0.5904 |
| Immune response | Integrin beta-1B | F (1, 20) =0.0008  P = 0.9776 | F (1, 20) =1.759  P = 0.1997 | F (1, 20) =0.451  P = 0.5095 |
|  | TNF | **F (1, 20) =11.79**  **P = 0.0026** | F (1, 20) =0.0529  P = 0.8203 | F (1, 20) =0.003  P = 0.9548 |

1. Author for correspondence (Email: jmzhao@yic.ac.cn) [↑](#footnote-ref-1)
